# Prognostic value of contrast staining on dual-energy CT after endovascular therapy in acute ischemic stroke: a meta-analysis

**DOI:** 10.1186/s12883-023-03370-9

**Published:** 2023-09-12

**Authors:** Fan Yang, Yi Zeng, Fei Zhu, Xiaoyan Hu

**Affiliations:** 1 Department of Radiology, Chengdu First People’s Hospital, Chengdu, Sichuan 610041 China; 2 Department of Radiology, Sichuan Province Orthopedic Hospital, Chengdu, Sichuan 610041 China; 3 Department of Radiology, West China Hospital, Sichuan University, Chengdu, Sichuan 610041 China

**Keywords:** Contrast staining, Dual-energy computed tomograpy, Acute ischemic stroke, Endovascular therapy

## Abstract

**Background:**

Contrast staining (CS) on dual-energy CT (DECT) is common after endovascular therapy (EVT) in acute ischemic stroke (AIS). We performed a meta-analysis to investigate the prognostic significance of CS detected by DECT after EVT in AIS.

**Method:**

MEDLINE, EMBASE, Cochrane Central Register of Controlled Trials, Web of Science and Scopus databases were searched from inception to July 2023 for publications on the prognostic significance of CS on DECT after EVT in patients with AIS. Prognostic outcomes were hemorrhage transformation (HT) and poor functional outcome (modified Rankin Scale [mRS] Score of 3–6 at the 90-day follow-up). Data are presented as odds ratios (OR) and 95% confidence intervals (CI).

**Results:**

Eleven studies including 1123 cases of AIS were included. Pooled results indicated a higher risk of HT in patients with CS than in those without CS (OR = 2.22; 95% CI 1.41–3.51, *P* = 0.001; *I*^*2*^ = 45.4%). No association between CS and symptomatic HT was observed (OR = 2.10; 95% CI 0.64–6.95, *P* = 0.223; *I*^*2*^ = 67.3%). Moreover, there was also higher odds of poor functional outcome in patients with CS than in those without CS (OR = 2.76; 95% CI 1.53–4.97, P = 0.001; *I*^*2*^ = 44.9%).

**Conclusions:**

The presence of contrast staining on DECT after EVT is associated with a higher risk of hemorrhage transformation and poor functional outcome. However, further high-quality studies with standardized processes are required to confirm these results.

**Supplementary Information:**

The online version contains supplementary material available at 10.1186/s12883-023-03370-9.

## Introduction

Acute ischemic stroke (AIS) is associated with high morbidity and mortality [1]. Endovascular therapy (EVT) is an effective therapeutic strategy for AIS caused by large-vessel occlusion [2, 3]. Administration of contrast material during EVT often results in hyperdense areas (HDA) on postprocedural brain non-contrast computed tomography (NCCT) related to blood-brain barrier (BBB) disruption [4]. Hyperdensity may be a sign of contrast staining (CS) or a combination of hemorrhage. CS usually resolves within 24–48 h [5]. However, intracranial hemorrhage is potentially associated with poor prognosis [6]. Although CS is also suggestive of ischemic injury to local vessels, anticoagulant therapy does not need to be discontinued as with hemorrhage. In the absence of intracranial hemorrhage, if anticoagulant therapy is abruptly interrupted, the previous therapeutic effect of thrombolysis may come to naught. Therefore, the identification of these hyperdense areas on CT as CS or hemorrhage as early as possible is clinically critical and significant.

It is difficult to differentiate contrast material from hemorrhage by using conventional single-energy CT scans. Dual-energy CT (DECT) has been shown to accurately differentiate intracranial hemorrhage from CS with excellent accuracy and specificity [7]. Furthermore, compared with patients without HDA (No-HDA), patients with CS have a longer onset-to-reperfusion time, and more numbers of thrombectomy devices pass as breaking the BBB [8–10]. Thus, late hemorrhage transformation (HT) may also occur in patients without signs of bleeding on CT immediately after EVT.

Several meta-analyses have been published on associations between HDA detected by CT and the clinical outcomes of EVT in patients with AIS [11, 12]. But no meta-analysis has been published with DECT only. By including studies containing conventional computed tomography, the diagnostic accuracy for CS may be reduced. Therefore, this meta-analysis aimed to investigate the association between CS detected by DECT after EVT and HT, as well as poor functional outcome. We hypothesized that CS detected by DECT may be associated with HT and poor functional outcome, which could provide some assistance in balancing the risks and benefits of antithrombotic therapy strategies for patients after EVT.

## Method

### Literature search strategy

This systematic review has been written with reference to the screening guidelines of the preferred reporting items for systematic reviews and meta-analyses (PRISMA).

This protocol was registered in the International Prospective Register of Systematic Reviews database (PROSPERO, registration number: CRD42022378646). Two authors (Y.F. and Z.Y.) with 11 years of radiological expertise separately searched the MEDLINE (PubMed), EMBASE (OvidSP), Cochrane Central Register of Controlled Trials (CENTRAL), Web of Science and Scopus databases through July 15, 2023, using the following keywords: Dual-Energy Head CT OR Dual Energy Computed Tomography, Cerebral Parenchymal Hemorrhages OR Intracranial Hemorrhage, Contrast Staining OR Iodine Contrast Extravasation OR Contrast Extravasation). The language of the studies was without any restriction. The references included in the studies were also searched to identify additional studies. Detailed search strategies are available in Supplementary Table 1.

### Inclusion and exclusion criteria

Two reviewers (Y.F. and Z.Y.) independently selected eligible primary studies and the disagreements were resolved by consensus. The inclusion criteria were as follows: (a) in which patients who received EVT after AIS; (b) in which patients who received contrast medium during EVT; (c) in which DECT was performed after EVT; (d) in which CT and/or magnetic resonance imaging (MRI) were as reference standards following DECT; (e) in which 90-day follow-up records were available; and (f) that contained sufficient data to construct 2 × 2 contingency tables or direct odds ratio (OR) values.

Duplications, irrelevant articles, animal studies, editorials, comments, correspondence, letters, case reports, conference abstracts, reviews, and meta-analyses were excluded.

### Data extraction and quality assessment

Two review authors (Y.F. and Z.Y.) independently extracted the following data from each included study: first author’s name, publication year, country, study design, number of cases, mean age, proportion of males, baseline NIHSS score, proportion of anterior circulation, proportion of successful recanalization, DECT protocols and time interval, reference standards and time interval, DECT numerical information (cases of CS, No-HDA, hemorrhage; as well as cases of HT and symptomatic HT (sHT) according to the criteria of ECASS or the Heidelberg bleeding classification [13, 14]), and cases of patients with poor outcome, which was defined as a modified Rankin Scale (mRS) score of 3–6 at the 90-day follow-up. Disagreements were resolved through consensus.

Two authors (Y.F. and Z.Y.) separately assessed the quality of each eligible study according to Newcastle-Ottawa Quality Assessment Scale for nonrandomized studies [15]. Case-control studies and cohort studies were assessed based on 3 aspects: patient selection, study comparability, and exposure or outcome. Disagreements were resolved by the two authors through discussions or by consulting a third author (H.X.Y).

### Statistical analysis

Stata V.15 software (StataCorp LP, College Station, Texas) was used for the combined statistical analysis. Data synthesis of the association between CS and HT/sHT, as well as poor outcome, are presented as OR and 95% CI. A random effects model was used for the meta-analysis. Statistical significance was set at *P* ≤ 0.05. Heterogeneity was assessed by using Cochrane Q statistics and was quantified via *I*^*2*^ with values 25–50%, 50–75%, and > 75% consistent with low, moderate, and high heterogeneity, respectively. Subgroup analysis was conducted based on the location of the lesion (anterior or posterior stroke) and DECT time interval (within 1 h or longer). Egger’s test was performed to detect publication bias.

## Results

### Study selection

**Fig. 1:**
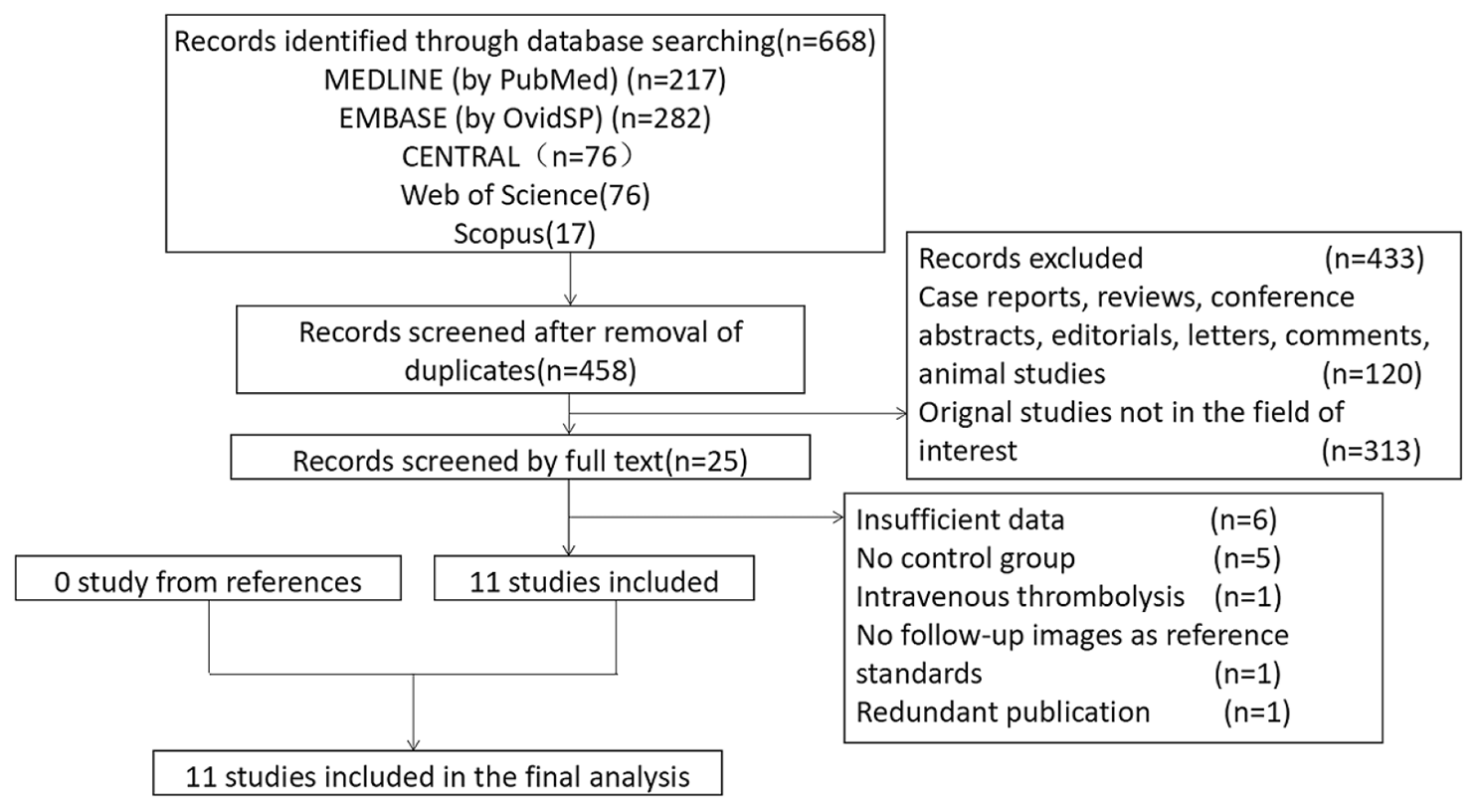
Flow diagram of the search strategy

The flow diagram of the search strategy is shown in Fig. 1. A total of 668 articles were identified using this search strategy. After screening titles and abstracts, 643 items were excluded based on the inclusion and exclusion criteria. We read the full texts of the remaining 25 potentially eligible studies and excluded 14. Six studies had no available original data that could be extracted, while five studies had no control groups. One study evaluated patients who underwent intravenous thrombolysis after AIS. One study had no follow-up images as reference standards and one study was duplicate publication. Finally, a total of 11 studies were included in the final analysis [8–10, 16–23].

### Characteristics of the Studies

The characteristics of the included studies are shown in Table 1 and online Supplementary Tables 3–6. In total, 5 case-control studies and 6 cohort studies, including 1184 patients with AIS treated with EVT, in which 1123 patients underwent the DECT and follow-up CT and/or MRI, were included in the meta-analysis. The selected studies were conducted between 2014 and 2022 in 6 countries. Eventually, 46.1% of the patients (546/1184) were diagnosed with CS only. Of the 11 studies included, 10 studies outlined the correlation between CS and HT [8–10, 16–19, 21–23], along with four studies on sHT [8, 16, 19, 20], while four studies reported the relationship between CS and poor functional outcome [8, 10, 16, 20]. One of the studies [16] evaluated the anterior circulation stroke and the posterior circulation stroke separately, so we extracted the data as if it were two separate studies. The quality of the included studies, according to the Newcastle-Ottawa Quality Assessment Scale, is shown in Table 1 and online Supplementary Table 2.

**Table 1:** Characteristics of the studies included

Author	Year	Country	Mean Age(y)	Proportion of anterior circulation	Proportion of successful recanalization	DECT time interval	Reference standard/time interval	Sample Size(n)	Outcome	Scores of NOS scale/study design
An, H. [13]	2019	China	61.3	65.6%	83%	12–24 hs	NCCT, 72 ± 6 hs	180	①②③	8/cohort
Bonatti, M. [14]	2018	Italy	70	89.4%	64.7%	within 1 h	NCCT, 24 hs	85	①	6/case-control
Byrne, D. [15]	2020	Canada	70	100%	100%	within 1 h	NCCT, 24 hs or earlier	71	①	7/case-control
Cai, J. [16]	2021	China	71	100%	80.3%	immediately	NCCT or MRI, 24 ± 4 hs	147	①②	7/case-control
Chen, Z. [8]	2020	China	63.1	100%	81.9%	within 24 hs	NCCT, 72–120 hs	166	①②③	9/cohort
Liu, K. [10]	2021	China	72	84.9%	84%	immediately	DECT, 24 hs	106	①③	7/cohort
Ma, C. [17]	2021	China	70	100%	100%	within 1 h	NCCT,48 hs	102	②③	7/case-control
Ma, C. [18]	2022	China	69	100%	100%	within 1 h	NCCT, 24- to 8- hs	138	①	7/case-control
Renú, A. [9]	2015	Spain	68	83%	84.5%	within 24 hs	NCCT or MRI, 48 to 72 hs	71	①	7/cohort
Tijssen, M. P. [19]	2014	Netherlands	56	na	na	immediately	NCCT, 24–48 hs	22	①	7/cohort
Zaouak, Y. [20]	2020	Belgium	55	na	na	within 1 h	NCCT or MRI, 24–48 hs	35	①	7/cohort

CT, computed tomography; DECT, dual energy computed tomography; NCCT, noncontrast computed tomography; NOS, Newcastle-Ottawa Scale; na, not available; ① = HT, hemorrhage transformation; ② = sHT, symptomatic HT; ③ = Modified Rankin Scale Score 3–6 at 90-day follow-up

### Relationships between CS after EVT and HT, sHT

**Fig. 2:**
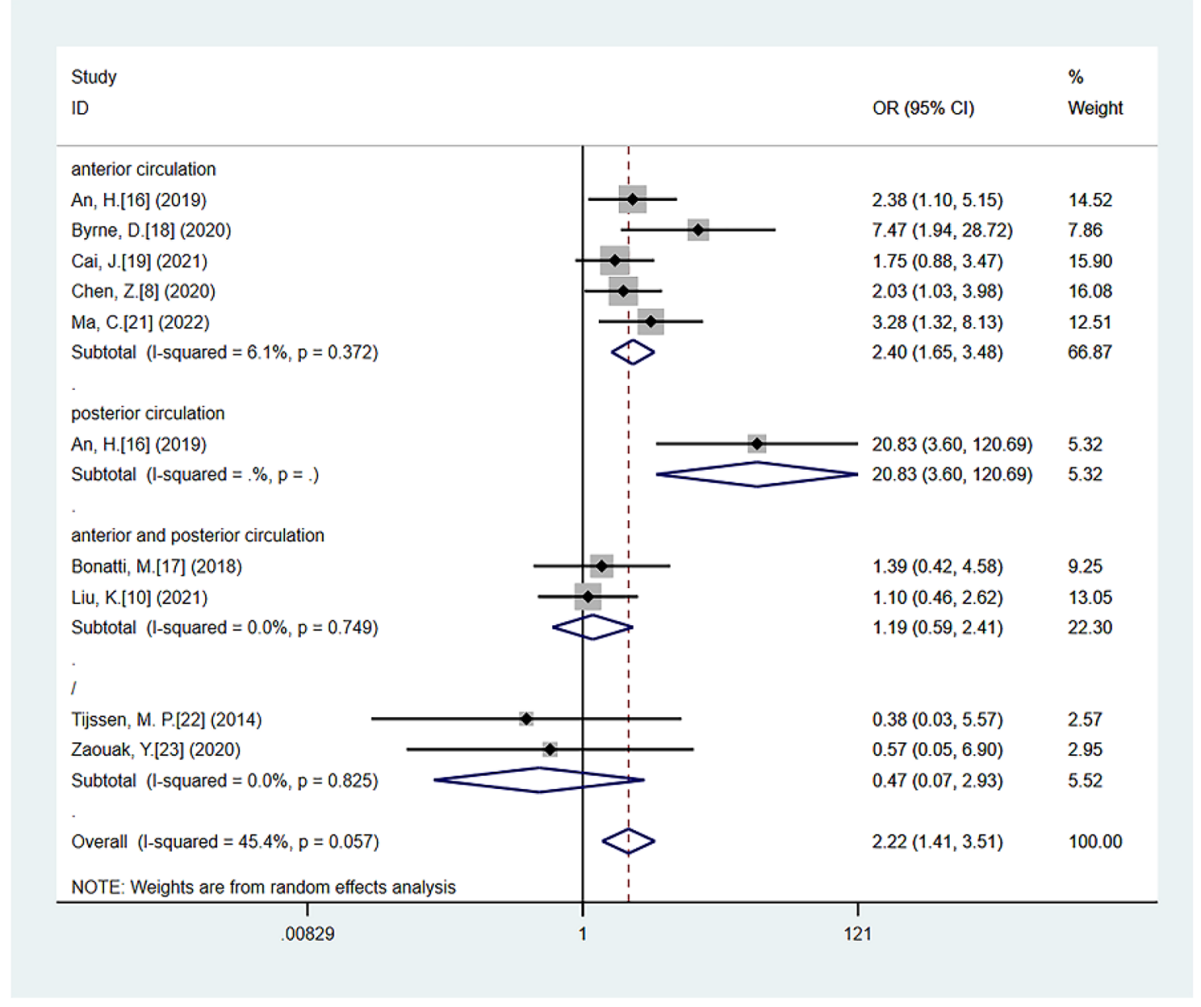
The forest plot of the relationships between CS and HT compared with Non-CS, including anterior circulation stroke subgroup analysis

**Fig. 3:**
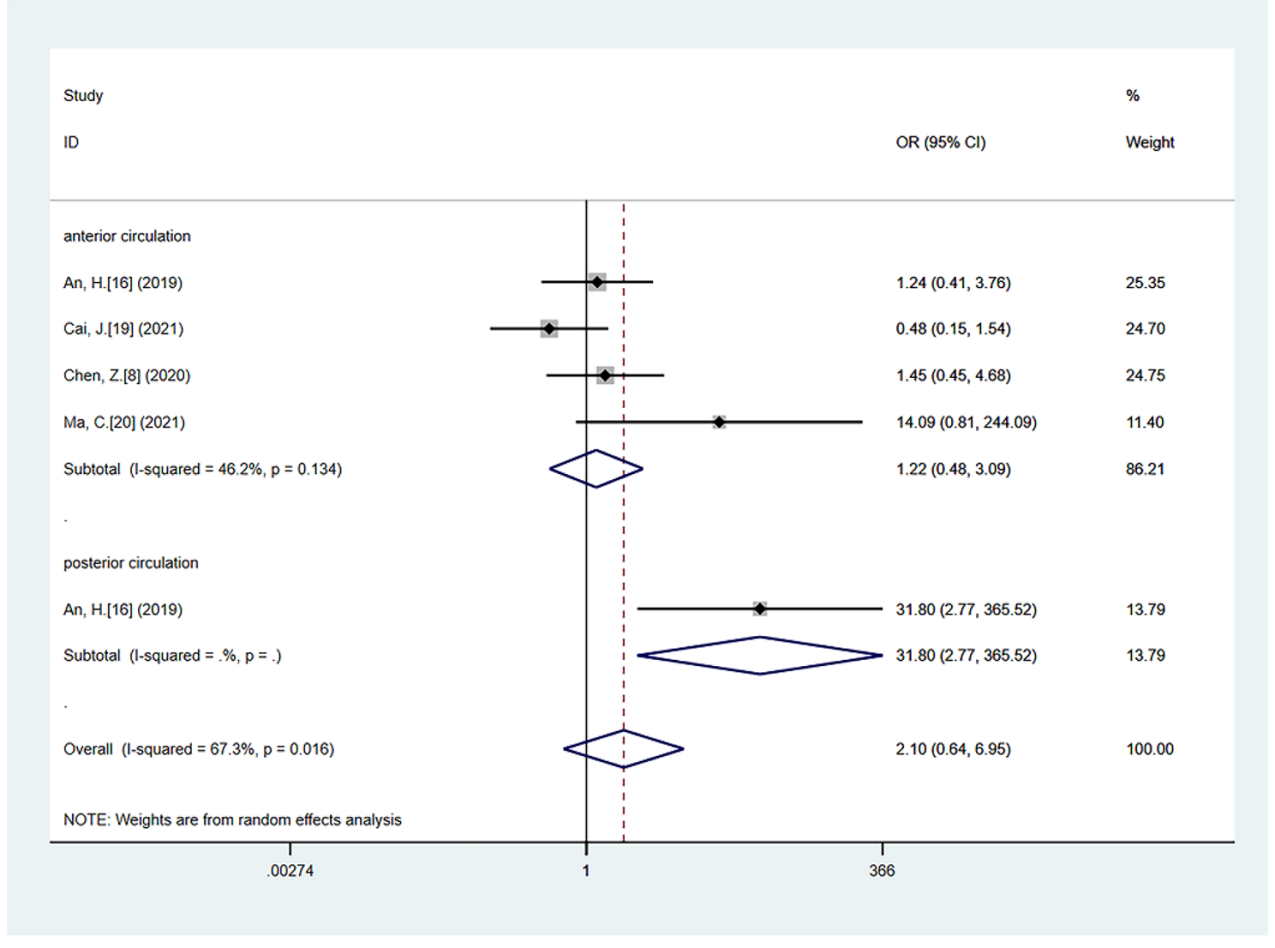
The forest plot of the relationships between CS and sHT compared with Non-CS, including anterior circulation stroke subgroup analysis

Compared with Non-CS patients, CS significantly increased the risk of HT (OR = 2.22; 95% CI 1.41–3.51; *P* = 0.001), with moderate heterogeneity (*P* = 0.057, *I*^*2*^ = 45.4%) [8, 10, 16–19, 21–23]. The association between CS and HT was still significant in the subgroup analysis, including studies that had underwent the index test time interval within 1 h (OR = 1.86; 95% CI: 1.07–3.23; *P* = 0.028; test of heterogeneity: *P* = 0.146, *I*^*2*^ = 37.0%) [10, 17–19, 21–23] and studies with anterior circulation stroke (OR = 2.40; 95% CI: 1.66–3.48; *P* < 0.001; test of heterogeneity: *P* = 0.372, *I*^*2*^ = 6.1%) [8, 15, 17, 18, 20] (Fig. 2). However, no association was found between CS and sHT (OR = 2.10; 95% CI 0.64–6.95; *P* = 0.223), with significant heterogeneity (*P* = 0.016, *I*^*2*^ = 67.3%) [8, 16, 19, 20]. Moreover, no association was observed between CS and sHT in the anterior circulation stroke subgroup (OR = 1.22; 95% CI: 0.48–3.09; *P* = 0.679; test of heterogeneity: *P* = 0.134, *I*^*2*^ = 46.2%) [8, 16, 19, 20] (Fig. 3).

Patients with CS had a higher incidence of HT at follow-up (OR = 6.56; 95% CI: 3.39–12.69; *P* < 0.001; test of heterogeneity: *P* = 0.307, *I*^*2*^ = 15.7%) than No-HDA patients [9, 10, 17–19, 21–23].

The Egger’s test showed that the outcomes of HT and sHT did not show any publication bias (see online Supplementary Tables 3–5).

### Relationships between CS after EVT and poor functional outcome

**Fig. 4:**
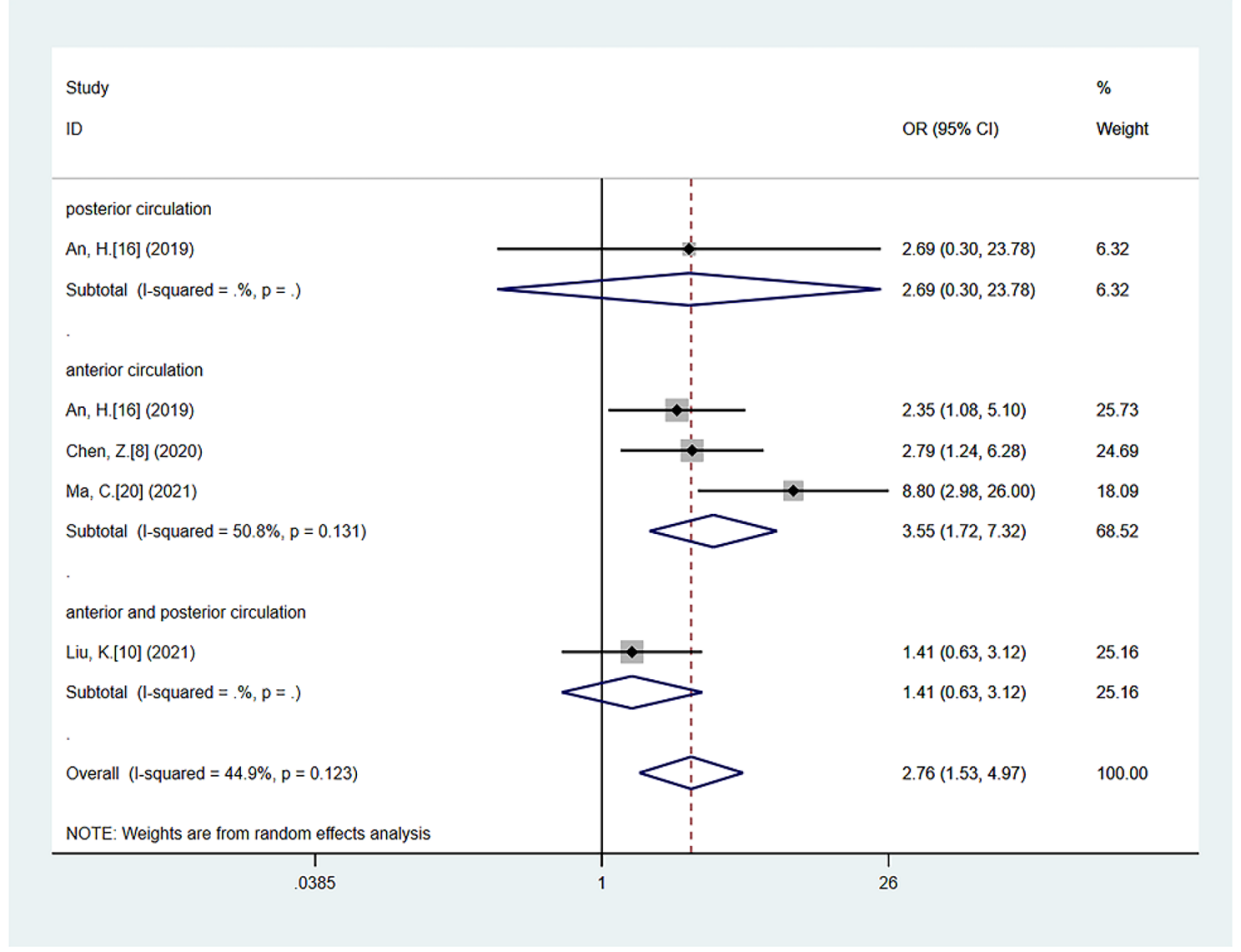
The forest plot of the relationships between CS and poor functional outcome compared with Non-CS, including anterior circulation stroke subgroup analysis

A meta-analysis of four eligible studies [8, 10, 16, 20] including 554 patients illustrated that patients with CS had a higher rate of poor functional outcome at 90-day follow-up than those without CS (OR = 2.76; 95% CI 1.53–4.97; P = 0.001), without heterogeneity (*P* = 0.123, *I*^*2*^ = 44.9%). The association between CS and poor outcome was still significant in the subgroup analysis, including studies on anterior circulation stroke (OR = 3.55; 95% CI: 1.72–7.32; *P* = 0.001; test of heterogeneity: *P* = 0.131, *I*^*2*^ = 50.8%) [8, 16, 20]. (Fig. 4)

Using the Egger’s test, no publication bias could be attributed to the poor functional outcome at the 90-day follow-up (see online Supplementary Table 6).

## Discussion

This meta-analysis aimed to investigate the prognostic significance of contrast staining detected by DECT after EVT. We identified that CS significantly increased the risk of HT and poor functional outcome at the 90-day follow-up in patients with acute ischemic stroke. However, no association was found between CS and sHT.

The recent advent of DECT has allowed for differentiation between contrast staining and hemorrhage following thrombectomy. Our study revealed that the presence of CS on DECT post-thrombectomy is probably associated with the development of HT, which supports the concept that both CS and HT are successive stages of BBB disruption [24]. Ahn et al. [25] showed that delayed petechial hemorrhage was predicted by anticoagulant use and maximum contrast density. It is recommended that the patients with evident CS following thrombectomy warrant close monitoring and necessary interventions such as avoiding or reversing anticoagulation [25].

Extravascular contrast material demonstrates washout within 24–48 h in a hyperdense area [5]. Thus, DECT should be performed as early as possible to detect CS post-EVT, and follow-up examinations are better after 24 h. If follow-up imaging is performed within 24 h after EVT, confusion will occur between CS and HT. In our study, the time interval between EVT and DECT differed from immediately [10, 17–23] to within 24 h [8, 9, 16] in different studies. Thus, we conducted a subgroup analysis of studies with time intervals within 1 h to reduce heterogeneity.

Various other possible covariates, including image analysis, location of the lesion, reference standard judgment, and the time of follow-up imaging, had a significant influence on the heterogeneity [7, 12]. Previous studies have identified that the posterior circulation has greater ischemic tolerance, and BBB disruption may be delayed after stroke onset compared with the anterior circulation [26, 27]. At the same time, the vasoreactivity and autoregulation of the anterior and posterior circulation are also different. In our study, the association between CS and HT was still significant in the subgroup analysis with studies on anterior circulation stroke without heterogeneity.

However, no association was found between CS and sHT, particularly in the anterior circulation stroke subgroup. A previous study indicated that a hyperdense area on CT after thrombectomy is significantly associated with a high risk of symptomatic intracerebral hemorrhage [12]. The opposite conclusion may be mainly attributed to the fact that high-density lesions after EVT also contain intracranial hemorrhage, which is considered to be a strong predictor of later sHT development [20].

We came up with a clinically significant result that CS after EVT was likely related to poor functional outcome. However, it was contrary to previous An’s [16] study. It may caused by the small sample size, various biases and random errors of the individual study. In addition, we specifically pooled patients with only anterior circulation stroke and found that CS presentation was associated with a 3.55-fold increase in the odds of a poor outcome. However, the exact mechanism of CS for poor outcome remains unclear. The assumptions for the mechanism are as follows. First, the contrast medium may cause neurotoxic effects via the inherent chemotoxicity [28], and allergic reactions as the contrast medium remains in the brain. Similarly, other studies have indicated that the use of contrast media is related to the deterioration of neurological function and conditions, such as posterior reversible leukoencephalopathy [29] and iodinated contrast encephalopathy [30]. Second, the risk of HT was increased in patients with CS on DECT, as mentioned above, and was associated with high mortality rates and significantly poor prognosis [31, 32]. A delayed reperfusion time and hyperperfusion after EVT may cause greater injury to the vasculature and BBB, further leading to evident CS [9]. Furthermore, the numbers of device passes per procedure and inappropriate operations during EVT may promote BBB disruption [9].

Our meta-analysis has several limitations. First, most of the included studies were retrospective and case-control, and patient selection could have introduced some bias. Second, the baseline NIHSS score, location of occlusion, proportion of successful recanalization, time interval of index test, time interval of reference standard and the DECT protocols were not the same among the included studies, which may have caused heterogeneity. However, we reduced this influence by performing a series of subgroup analysis. Third, although CS could predict HT in the subgroup analysis of studies that had an index test time interval within 1 h, we need to be aware that the lower limit of the confidence interval for OR was 1.07, but may fluctuate to the left or right due to a new research, and might further change our results.

## Conclusion

Our results indicate that the presence of contrast staining on DECT after endovascular therapy for acute ischemic stroke is probably associated with a high risk of hemorrhage transformation and poor functional outcome. However, our assessment of the evidence has several limitations. Further high-quality studies with standardized processes are required to validate our findings.

### Electronic supplementary material

Supplementary material

## Data Availability

All data generated or analysed during this study are included in this published article [and its supplementary information files].

## Abbreviations

CS: contrast staining
DECT: dual-energy CT
EVT: endovascular therapy
AIS: acute ischemic stroke
HT: hemorrhage transformation
mRS: modified Rankin Scale
OR: odds ratio
CI: confidence interval
HDA: hyperdense area
NCCT: non-contrast computed tomography
BBB: blood-brain barrier

## References

[1] Benjamin EJ, Virani SS, Callaway CW, Chamberlain AM, Chang AR, Cheng S (2018). Heart Disease and Stroke Statistics-2018 update: a Report from the American Heart Association [published correction appears in circulation. 2018;137(12):e493]. Circulation.

[2] Berkhemer OA, Fransen PS, Beumer D, van den Berg LA, Lingsma HF, Yoo AJ (2015). A randomized trial of intraarterial treatment for acute ischemic stroke [published correction appears in N Engl J Med. 2015;372(4):394]. N Engl J Med.

[3] Goyal M, Menon BK, van Zwam WH, Dippel DW, Mitchell PJ, Demchuk AM (2016). Endovascular thrombectomy after large-vessel ischaemic stroke: a meta-analysis of individual patient data from five randomised trials. Lancet.

[4] Lummel N, Schulte-Altedorneburg G, Bernau C, Pfefferkorn T, Patzig M, Janssen H (2014). Hyperattenuated intracerebral lesions after mechanical recanalization in acute stroke. AJNR Am J Neuroradiol.

[5] Nikoubashman O, Reich A, Gindullis M, Frohnhofen K, Pjontek R, Brockmann MA (2014). Clinical significance of post-interventional cerebral hyperdensities after endovascular mechanical thrombectomy in acute ischaemic stroke. Neuroradiology.

[6] Zhang X, Xie Y, Wang H, Yang D, Jiang T, Yuan K (2020). Symptomatic intracranial hemorrhage after mechanical thrombectomy in chinese ischemic stroke patients: the ASIAN score. Stroke.

[7] Chen S, Zhang J, Quan X, Xie Y, Deng X, Zhang Y (2022). Diagnostic accuracy of dual-energy computed tomography to differentiate intracerebral hemorrhage from contrast extravasation after endovascular thrombectomy for acute ischemic stroke: systematic review and meta-analysis. Eur Radiol.

[8] Chen Z, Zhang Y, Su Y, Sun Y, He Y, Chen H (2020). Contrast Extravasation is predictive of poor clinical outcomes in patients undergoing endovascular therapy for Acute ischemic stroke in the anterior circulation. J Stroke Cerebrovasc Dis.

[9] Renú A, Amaro S, Laredo C, Román LS, Llull L, Lopez A (2015). Relevance of blood-brain barrier disruption after endovascular treatment of ischemic stroke: dual-energy computed tomographic study. Stroke.

[10] Liu K, Jiang L, Zhao Y, Xia W, Ruan J, Huang H (2021). Risk factors of contrast extravasation and subsequent hemorrhagic transformation after thrombectomy. J Int Med Res.

[11] Xu T, Wang Y, Yuan J, Chen Y, Luo H (2021). Contrast extravasation and outcome of endovascular therapy in acute ischaemic stroke: a systematic review and meta-analysis. BMJ Open.

[12] Jiang Q, Hou J, Ge J, Huang Z, Wang H, Guo Z (2021). Clinical significance of Hyperdense Area after Endovascular Therapy in patients with Acute ischemic stroke: a systematic review and Meta-analysis. Cerebrovasc Dis.

[13] Larrue V, von Kummer R, del Zoppo G, Bluhmki E (1997). Hemorrhagic transformation in acute ischemic stroke. Potential contributing factors in the European Cooperative Acute Stroke Study. Stroke.

[14] von Kummer R, Broderick JP, Campbell BC, Demchuk A, Goyal M, Hill MD (2015). The Heidelberg bleeding classification: classification of bleeding events after ischemic stroke and reperfusion therapy. Stroke.

[15] Stang A (2010). Critical evaluation of the Newcastle-Ottawa scale for the assessment of the quality of nonrandomized studies in meta-analyses. Eur J Epidemiol.

[16] An H, Zhao W, Wang J, Wright JC, Elmadhoun O, Wu D (2019). Contrast staining may be Associated with Intracerebral Hemorrhage but not functional outcome in Acute ischemic stroke patients treated with endovascular thrombectomy. Aging Dis.

[17] Bonatti M, Lombardo F, Zamboni GA, Vittadello F, Currò Dossi R, Bonetti B (2018). Iodine extravasation quantification on dual-energy CT of the Brain Performed after Mechanical Thrombectomy for Acute Ischemic Stroke can predict hemorrhagic complications. AJNR Am J Neuroradiol.

[18] Byrne D, Walsh JP, Schmiedeskamp H, Settecase F, Heran MKS, Niu B (2020). Prediction of hemorrhage after successful recanalization in patients with Acute Ischemic Stroke: Improved Risk Stratification using dual-energy CT parenchymal iodine concentration ratio relative to the Superior Sagittal Sinus. AJNR Am J Neuroradiol.

[19] Cai J, Zhou Y, Zhao Y, Xu C, Yan S, Ding X (2021). Comparison of various reconstructions derived from dual-energy CT immediately after endovascular treatment of acute ischemic stroke in predicting hemorrhage. Eur Radiol.

[20] Ma C, Hui Q, Gao X, Xu D, Tang B, Pen M (2021). The feasibility of dual-energy CT to predict the probability of symptomatic intracerebral haemorrhage after successful mechanical thrombectomy. Clin Radiol.

[21] Ma C, Xu D, Hui Q, Gao X, Peng M (2022). Quantitative Intracerebral Iodine Extravasation in Risk Stratification for Intracranial Hemorrhage in patients with Acute ischemic stroke. AJNR Am J Neuroradiol.

[22] Tijssen MP, Hofman PA, Stadler AA, van Zwam W, de Graaf R, van Oostenbrugge RJ (2014). The role of dual energy CT in differentiating between brain haemorrhage and contrast medium after mechanical revascularisation in acute ischaemic stroke. Eur Radiol.

[23] Zaouak Y, Sadeghi N, Sarbu N, Ligot N, Lubicz B (2020). Differentiation between cerebral hemorrhage and contrast Extravasation using Dual Energy Computed Tomography after Intra-Arterial Neuro Interventional Procedures. J Belg Soc Radiol.

[24] Nakano S, Iseda T, Kawano H, Yoneyama T, Ikeda T, Wakisaka S (2001). Parenchymal hyperdensity on computed tomography after intra-arterial reperfusion therapy for acute middle cerebral artery occlusion: incidence and clinical significance. Stroke.

[25] Ahn S, Roth SG, Mummareddy N, Ko Y, Bhamidipati A, Jo J (2023). The clinical utility of dual-energy CT in post-thrombectomy care: part 2, the predictive value of contrast density and volume for delayed hemorrhagic transformation [published online ahead of print, 2023 Jun 29]. J Stroke Cerebrovasc Dis.

[26] Lee M, Saver JL, Alger JR, Hao Q, Starkman S, Ali LK (2012). Blood-brain barrier permeability derangements in posterior circulation ischemic stroke: frequency and relation to hemorrhagic transformation. J Neurol Sci.

[27] Menon BK, O’Brien B, Bivard A, Spratt NJ, Demchuk AM, Miteff F (2013). Assessment of leptomeningeal collaterals using dynamic CT angiography in patients with acute ischemic stroke. J Cereb Blood Flow Metab.

[28] Gomi N (1992). Vasoconstriction by angiographic contrast media in isolated canine arteries. Br J Radiol.

[29] Saigal G, Bhatia R, Bhatia S, Wakhloo AK (2004). MR findings of cortical blindness following cerebral angiography: is this entity related to posterior reversible leukoencephalopathy?. AJNR Am J Neuroradiol.

[30] Leong S, Fanning NF (2012). Persistent neurological deficit from iodinated contrast encephalopathy following intracranial aneurysm coiling. A case report and review of the literature. Interv Neuroradiol.

[31] Paciaroni M, Agnelli G, Corea F, Ageno W, Alberti A, Lanari A (2008). Early hemorrhagic transformation of brain infarction: rate, predictive factors, and influence on clinical outcome: results of a prospective multicenter study. Stroke.

[32] Rao NM, Levine SR, Gornbein JA, Saver JL (2014). Defining clinically relevant cerebral hemorrhage after thrombolytic therapy for stroke: analysis of the National Institute of Neurological Disorders and Stroke tissue-type plasminogen activator trials. Stroke.

