# Supplementary material for: Prognostic value of contrast staining on dual-energy CT after endovascular therapy in acute ischemic stroke: a meta-analysis
Source: BMC Neurol. 2023 Sep 12;23:326. doi: 10.1186/s12883-023-03370-9 (PMC10496411; doi:10.1186/s12883-023-03370-9)
Supplement: Supplementary file 1 — Supplementary material [file 12883_2023_3370_MOESM1_ESM.doc]

Prognostic value of contrast staining on DECT in acute ischemic stroke after endovascular therapy: A meta-analysis

Fan Yang^1^, Yi Zeng^2^, Fei Zhu^3^, Xiaoyan Hu^1^

1Department of Radiology, Chengdu First People’s Hospital, Chengdu 610041, Sichuan, China.

2Department of Radiology, Sichuan Province Orthopedic Hospital, Chengdu 610041, Sichuan, China.

3Department of Radiology, West China Hospital, Sichuan University, Chengdu 610041, Sichuan, China.

Corresponding Author:

Xiaoyan Hu, MD

Department of Radiology, Chengdu First People’s Hospital, Chengdu 610041, Sichuan, China

Tel: 86-15184456293

E-mail: [hxy6293@foxmail.com](mailto:hxy6293@foxmail.com)

Supplementary table 1: Detailed MEDLINE, EMBASE and CENTRAL databases search strategy for article selection.

| Database | Step | Search algorithm | Items found |
| --- | --- | --- | --- |
| PubMed Medline | #1  #2  #3  #4  #5 | (((DECT[Title/Abstract]) OR (Dual-Energy CT[Title/Abstract])) OR (Dual-Energy Head CT[Title/Abstract])) OR (Dual Energy Computed Tomography [Title/Abstract])  (("Cerebral Hemorrhage"[Mesh]) OR (((((((((((((((((((((((Hemorrhage, Cerebrum[Title/Abstract]) OR (Cerebrum Hemorrhage[Title/Abstract])) OR (Cerebrum Hemorrhages[Title/Abstract])) OR (Hemorrhages, Cerebrum[Title/Abstract])) OR (Cerebral Parenchymal Hemorrhage[Title/Abstract])) OR (Cerebral Parenchymal Hemorrhages[Title/Abstract])) OR (Hemorrhage, Cerebral Parenchymal[Title/Abstract])) OR (Hemorrhages, Cerebral Parenchymal[Title/Abstract])) OR (Parenchymal Hemorrhage, Cerebral[Title/Abstract])) OR (Parenchymal Hemorrhages, Cerebral[Title/Abstract])) OR (Intracerebral Hemorrhage[Title/Abstract])) OR (Hemorrhage, Intracerebral[Title/Abstract])) OR (Hemorrhages, Intracerebral[Title/Abstract])) OR (Intracerebral Hemorrhages[Title/Abstract])) OR (Hemorrhage, Cerebral[Title/Abstract])) OR (Cerebral Hemorrhages[Title/Abstract])) OR (Hemorrhages, Cerebral[Title/Abstract])) OR (Brain Hemorrhage, Cerebral[Title/Abstract])) OR (Brain Hemorrhages, Cerebral[Title/Abstract])) OR (Cerebral Brain Hemorrhage[Title/Abstract])) OR (Cerebral Brain Hemorrhages[Title/Abstract])) OR (Hemorrhage, Cerebral Brain[Title/Abstract])) OR (Hemorrhages, Cerebral Brain[Title/Abstract]))  ((((((((((contrast extravasates [Title/Abstract]) OR (Iodine contrast extravasates [Title/Abstract])) OR (contrast extravasate [Title/Abstract])) OR (Iodinated Contrast Material Staining [Title/Abstract])) OR (Iodinated Contrast [Title/Abstract])) OR (contrast medium [Title/Abstract])) OR (Iodine Extravasation Quantification [Title/Abstract])) OR (Extravasation of Diagnostic, Therapeutic Materials [Title/Abstract])) OR (Extravasation of Contrast Media [Title/Abstract])) OR (Contrast Media  Extravasation [Title/Abstract])) OR ("Extravasation of Diagnostic and Therapeutic Materials"[Mesh])))  #2 OR #3  #1 AND #4 | 3,770  48,394  22,321  70,514  217 |
| Embase | #1  #2  #3  #4  #5 | 'dect' OR 'dual-energy ct' OR 'dual-energy head ct'.ab,ti  'Hemorrhage, Cerebrum' OR 'Cerebrum Hemorrhage' OR 'Cerebrum Hemorrhages' OR 'Hemorrhages, Cerebrum' OR 'Cerebral Parenchymal Hemorrhage' OR 'Cerebral Parenchymal Hemorrhages' OR 'Hemorrhage, Cerebral Parenchymal' OR 'Hemorrhages, Cerebral Parenchymal' OR 'Parenchymal Hemorrhage, Cerebral' OR 'Parenchymal Hemorrhages, Cerebral' OR 'Intracerebral Hemorrhage' OR 'Hemorrhage, Intracerebral' OR 'Hemorrhages, Intracerebral' OR 'Intracerebral Hemorrhages' OR 'Hemorrhage, Cerebral' OR 'Cerebral Hemorrhages' OR 'Hemorrhages, Cerebral' OR 'Brain Hemorrhage, Cerebral' OR 'Brain Hemorrhages, Cerebral' OR 'Cerebral Brain Hemorrhage' OR 'Cerebral Brain Hemorrhages' OR 'Hemorrhage, Cerebral Brain' OR 'Hemorrhages, Cerebral Brain'.ab,ti  contrast extravasates OR Iodine contrast extravasates OR contrast extravasate OR Iodinated Contrast Material Staining OR Iodinated Contrast OR contrast medium OR Iodine Extravasation Quantification OR Extravasation of Diagnostic, Therapeutic Materials OR Extravasation of Contrast Media OR Contrast media Extravasation.ab,ti  #2 OR #3  #1 AND #4 | 5168  26509  23028  49486  282 |
| the Cochrane Central Register of Controlled Trials (CENTRAL) | #1  #2  #3  #4  #5  #6  #7  #8  #9 | (Dual Energy Computed Tomography): ab, ti, kw OR (DECT): ab, ti, kw OR (Dual-Energy CT): ab, ti, kw OR (Dual-Energy Head CT): ab, ti, kw  MeSH descriptor: [Cerebral Hemorrhage] explode all trees  (Hemorrhage, Cerebrum):ab,ti,kw OR (Cerebrum Hemorrhage):ab,ti,kw OR (Cerebrum Hemorrhages):ab,ti,kw OR (Hemorrhages, Cerebrum):ab,ti,kw OR (Cerebral Parenchymal Hemorrhage):ab,ti,kw OR (Cerebral Parenchymal Hemorrhages):ab,ti,kw OR (Hemorrhage, Cerebral Parenchymal):ab,ti,kw OR (Hemorrhages, Cerebral Parenchymal):ab,ti,kw OR (Parenchymal Hemorrhage, Cerebral):ab,ti,kw OR (Parenchymal Hemorrhages, Cerebral):ab,ti,kw OR (Intracerebral Hemorrhage):ab,ti,kw OR (Hemorrhage, Intracerebral):ab,ti,kw OR (Hemorrhages, Intracerebral):ab,ti,kw OR (Intracerebral Hemorrhages):ab,ti,kw OR (Hemorrhage, Cerebral):ab,ti,kw OR (Cerebral Hemorrhages):ab,ti,kw OR (Hemorrhages, Cerebral):ab,ti,kw OR (Brain Hemorrhage, Cerebral):ab,ti,kw OR (Brain Hemorrhages, Cerebral):ab,ti,kw OR (Cerebral Brain Hemorrhage):ab,ti,kw OR (Cerebral Brain Hemorrhages):ab,ti,kw OR (Hemorrhage, Cerebral Brain):ab,ti,kw OR (Hemorrhages, Cerebral Brain):ab,ti,kw  #2 OR #3  MeSH descriptor: [ Extravasation of Diagnostic and Therapeutic Materials] explode all trees  (contrast extravasates): ab, ti, kw OR (Iodine contrast extravasates): ab, ti, kw OR (contrast extravasate): ab, ti, kw OR (Iodinated Contrast Material Staining): ab, ti, kw OR (Iodinated Contrast): ab, ti, kw OR (contrast medium): ab, ti, kw OR (Iodine Extravasation Quantification): ab, ti, kw OR (Extravasation of Diagnostic, Therapeutic Materials): ab, ti, kw OR (Extravasation of Contrast Media): ab, ti, kw OR (Contrast Media Extravasation): ab, ti, kw  #5 OR #6  #4 OR #7  #1 AND #8 | 1313  1373  7050  7059  598  3795  4335  11372  76 |
| Web of Science | #1  #2  #3  #4 | (TS=dect) OR(TS=dual-energy ct) OR (TS=dual-energy head ct)  (TS=Hemorrhage, Cerebrum) OR (TS=Cerebrum Hemorrhage) OR (TS=Cerebrum Hemorrhages) OR (TS=Hemorrhages, Cerebrum) OR (TS=Cerebral Parenchymal Hemorrhage) OR (TS=Cerebral Parenchymal Hemorrhages) OR (TS=Hemorrhage, Cerebral Parenchymal) OR (TS=Hemorrhages, Cerebral Parenchymal) OR (TS=Parenchymal Hemorrhage, Cerebral) OR (TS=Parenchymal Hemorrhages, Cerebral) OR (TS=Intracerebral Hemorrhage) OR (TS=Hemorrhage, Intracerebral) OR (TS=Hemorrhages, Intracerebral) OR (TS=Intracerebral Hemorrhages) OR (TS=Hemorrhage, Cerebral) OR (TS=Cerebral Hemorrhages) OR (TS=Hemorrhages, Cerebral) OR (TS=Brain Hemorrhage, Cerebral) OR (TS=Brain Hemorrhages, Cerebral) OR (TS=Cerebral Brain Hemorrhage) OR (TS=Cerebral Brain Hemorrhages) OR (TS=Hemorrhage, Cerebral Brain) OR (TS=Hemorrhages, Cerebral Brain)  (TS=contrast extravasates) OR (TS=Iodine contrast extravasates) OR (TS=contrast extravasate) OR (TS=Iodinated Contrast Material Staining) OR (TS=Iodinated Contrast) OR (TS=contrast medium) OR (TS=Iodine Extravasation Quantification) OR (TS=Extravasation of Diagnostic, Therapeutic Materials) OR (TS=Extravasation of Contrast Media) OR (TS=Contrast media Extravasation)  #1 and #2 and #3 | 12,455  124,239  286,936  76 |
| Scopus | #1  #2  #3  #4 | TITLE-ABS-KEY (dect) OR TITLE-ABS-KEY (dual-energy AND ct) OR TITLE-ABS-KEY ( dual-energy AND head AND ct )  TITLE-ABS-KEY (Hemorrhage, Cerebrum) OR TITLE-ABS-KEY(Cerebrum Hemorrhage) OR TITLE-ABS-KEY(Cerebrum Hemorrhages) OR TITLE-ABS-KEY(Hemorrhages, Cerebrum) OR TITLE-ABS-KEY(Cerebral Parenchymal Hemorrhag) OR TITLE-ABS-KEY(Cerebral Parenchymal Hemorrhages) OR TITLE-ABS-KEY(Hemorrhage, Cerebral Parenchymal) OR TITLE-ABS-KEY(Hemorrhages, Cerebral Parenchymal) OR TITLE-ABS-KEY(Parenchymal Hemorrhage, Cerebral) OR TITLE-ABS-KEY(Parenchymal Hemorrhages, Cerebral) OR TITLE-ABS-KEY(Intracerebral Hemorrhage) OR TITLE-ABS-KEY(Hemorrhage, Intracerebral) OR TITLE-ABS-KEY(Hemorrhages, Intracerebral)' OR TITLE-ABS-KEY(Intracerebral Hemorrhages) OR TITLE-ABS-KEY(Hemorrhage, Cerebral) OR TITLE-ABS-KEY(Cerebral Hemorrhages) OR TITLE-ABS-KEY(Hemorrhages, Cerebral) OR TITLE-ABS-KEY(Brain Hemorrhage, Cerebral) OR TITLE-ABS-KEY(Brain Hemorrhages, Cerebral) OR TITLE-ABS-KEY(Cerebral Brain Hemorrhage) OR TITLE-ABS-KEY(Cerebral Brain Hemorrhages) OR TITLE-ABS-KEY(Hemorrhage, Cerebral Brain) OR TITLE-ABS-KEY(Hemorrhages, Cerebral Brain)  TITLE-ABS-KEY(contrast extravasates) OR TITLE-ABS-KEY(Iodine contrast extravasates) OR TITLE-ABS-KEY(contrast extravasate) OR TITLE-ABS-KEY(Iodinated Contrast Material Staining) OR TITLE-ABS-KEY(Iodinated Contrast OR contrast medium) OR TITLE-ABS-KEY(Iodine Extravasation Quantification) OR TITLE-ABS-KEY(Extravasation of Diagnostic, Therapeutic Materials) OR TITLE-ABS-KEY(Extravasation of Contrast Media) OR TITLE-ABS-KEY(Contrast media Extravasation)  #1 and #2 and #3 | 8,803  26,933  16,446  17 |

Supplementary table 2: The quality of the included studies according to the Newcastle-Ottawa Quality Assessment Scale.

| Author | Year | Study design | Selection | Comparability | Exposure/Outcome | Quality scores |
| --- | --- | --- | --- | --- | --- | --- |
| An, H. [1] | 2019 | cohort | 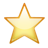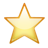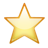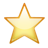 | 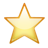 | 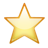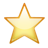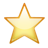 | 8 |
| Bonatti, M. [2] | 2018 | case-control | 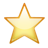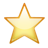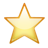 | 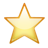 | 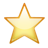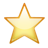 | 6 |
| Byrne, D. [3] | 2020 | case-control | 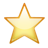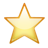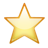 | 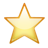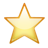 | 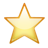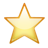 | 7 |
| Cai, J. [4] | 2021 | case-control | 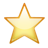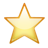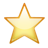 | 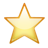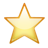 | 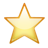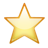 | 7 |
| Chen, Z. [5] | 2020 | cohort | 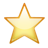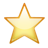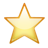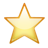 | 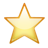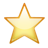 | 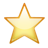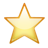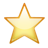 | 9 |
| Liu, K. [6] | 2021 | cohort | 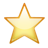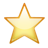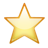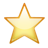 | 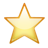 | 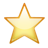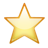 | 7 |
| Ma, C. [7] | 2021 | case-control | 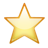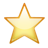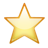 | 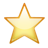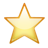 | 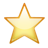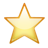 | 7 |
| Ma, C. [8] | 2022 | case-control | 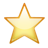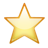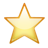 | 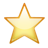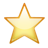 | 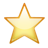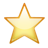 | 7 |
| Renú, A. [9] | 2015 | cohort | 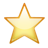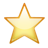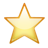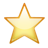 | 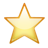 | 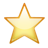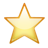 | 7 |
| Tijssen, M. P. [10] | 2014 | cohort | 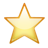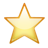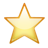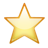 |  | 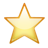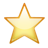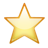 | 7 |
| Zaouak, Y. [11] | 2020 | cohort | 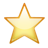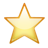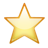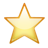 |  | 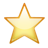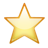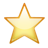 | 7 |

Supplementary table 3: Characteristics of included studies and the relationships between CS and HT compared with Non-CS.

|  | Sample size (n) | CS | | Non-CS | | Egger’s test (P) |
| --- | --- | --- | --- | --- | --- | --- |
|  |  | HT (n) | Non-HT (n) | HT (n) | Non-HT (n) |  |
| CS vs Non-CS |  |  |  |  |  | 0.31 |
| An, H. [1] | 180  (118)^*^ | 30  (25)^*^ | 20  (17)^*^ | 33  (29)^*^ | 97  (47)^*^ |  |
| Bonatti, M. [2] | 85 | 9 | 40 | 5 | 31 |  |
| Byrne, D. [3] | 71 | 17 | 22 | 3 | 29 |  |
| Cai, J. [4] | 147 | 35 | 20 | 46 | 46 |  |
| Chen, Z. [5] | 166 | 25 | 26 | 37 | 78 |  |
| Liu, K. [6] | 106 | 21 | 47 | 11 | 27 |  |
| Ma, C. [7] | 138 | 35 | 58 | 7 | 38 |  |
| Tijssen, M. P. [8] | 22 | 2 | 16 | 1 | 3 |  |
| Zaouak, Y. [9] | 35 | 1 | 15 | 2 | 17 |  |

*: anterior circulation stroke subgroup

Supplementary table 4: Characteristics of included studies and the relationships between CS and sHT.

|  | Sample size (n) | CS | | Non-CS | | Egger’s test (P) |
| --- | --- | --- | --- | --- | --- | --- |
|  |  | sHT (n) | Non-sHT (n) | sHT (n) | Non-sHT (n) |  |
| CS vs Non-CS |  |  |  |  |  | 0.06 |
| An, H.[1] | 180  (118)^*^ | 9  (6)^*^ | 41  (36)^*^ | 10  (9)^*^ | 120  (67)^*^ |  |
| Cai, J. [4] | 147 | 4 | 51 | 13 | 79 |  |
| Chen, Z. [5] | 166 | 5 | 46 | 8 | 107 |  |
| Ma, C. [10] | 102 | 15 | 60 | 0 | 27 |  |

*: anterior circulation stroke subgroup

Supplementary table 5: Characteristics of included studies and the relationships between CS and HT compared with No-HDA.

|  | Sample size (n) | CS | | No-HDA | | Egger’s test (P) |
| --- | --- | --- | --- | --- | --- | --- |
|  |  | HT (n) | Non-HT (n) | HT (n) | Non-HT (n) |  |
| CS vs No-HDA |  |  |  |  |  | 0.61 |
| Bonatti, M.[2] | 85 | 9 | 40 | 0 | 31 |  |
| Byrne, D.[3] | 71 | 17 | 22 | 0 | 28 |  |
| Cai, J.[4] | 147 | 35 | 20 | 5 | 37 |  |
| Liu, K.[6] | 106 | 21 | 47 | 0 | 27 |  |
| Ma, C. [7] | 138 | 35 | 58 | 5 | 38 |  |
| Renú, A.[11] | 71 | 10 | 17 | 7 | 37 |  |
| Tijssen, M. P.[8] | 22 | 2 | 16 | 0 | 3 |  |
| Zaouak, Y.[9] | 35 | 1 | 15 | 0 | 17 |  |

Supplementary table 6: Characteristics of included studies and the relationships between CS and poor functional outcome compared with Non-CS.

|  | Sample size (n) | CS | | Non-CS | | Egger’s test (P) |
| --- | --- | --- | --- | --- | --- | --- |
|  |  | Poor outcome (n) | Non-Poor outcome (n) | Poor outcome (n) | Non-Poor outcome (n) |  |
| CS vs Non-CS |  |  |  |  |  | 0.57 |
| An, H.[1] | 180  (118)^*^ | 34  (27)^*^ | 16  (15)^*^ | 72  (33)^*^ | 58  (43)^*^ |  |
| Chen, Z.[2] | 166 | 42 | 9 | 72 | 43 |  |
| Liu, K.[3] | 106 | 38 | 30 | 18 | 20 |  |
| Ma, C.[4] | 102 | 50 | 25 | 5 | 22 |  |

*: anterior circulation stroke subgroup

**References**

1. An H, Zhao W, Wang J, Wright JC, Elmadhoun O, Wu D, et al. Contrast Staining may be Associated with Intracerebral Hemorrhage but Not Functional Outcome in Acute Ischemic Stroke Patients Treated with Endovascular Thrombectomy. *Aging Dis*. 2019;10(4):784-792.
2. Bonatti M, Lombardo F, Zamboni GA, Vittadello F, Currò Dossi R, Bonetti B, et al. Iodine Extravasation Quantification on Dual-Energy CT of the Brain Performed after Mechanical Thrombectomy for Acute Ischemic Stroke Can Predict Hemorrhagic Complications. *AJNR Am J Neuroradiol*. 2018;39(3):441-447.
3. Byrne D, Walsh JP, Schmiedeskamp H, Settecase F, Heran MKS, Niu B, et al. Prediction of Hemorrhage after Successful Recanalization in Patients with Acute Ischemic Stroke: Improved Risk Stratification Using Dual-Energy CT Parenchymal Iodine Concentration Ratio Relative to the Superior Sagittal Sinus. *AJNR Am J Neuroradiol*. 2020;16.
4. Cai J, Zhou Y, Zhao Y, Xu C, Yan S, Ding X, et al. Comparison of various reconstructions derived from dual-energy CT immediately after endovascular treatment of acute ischemic stroke in predicting hemorrhage. *Eur Radiol*. 2021;31(7):4419-4427.41(1):64-70.
5. Chen Z, Zhang Y, Su Y, Sun Y, He Y, Chen H. Contrast Extravasation is Predictive of Poor Clinical Outcomes in Patients Undergoing Endovascular Therapy for Acute Ischemic Stroke in the Anterior Circulation. *J Stroke Cerebrovasc Dis*. 2020;29(1):104494.
6. Liu K, Jiang L, Zhao Y, Xia W, Ruan J, Huang H, et al. Risk factors of contrast extravasation and subsequent hemorrhagic transformation after thrombectomy. *J Int Med Res.* 2021;49(10):3000605211049074.
7. Ma C, Hui Q, Gao X, Xu D, Tang B, Pen M, et al. The feasibility of dual-energy CT to predict the probability of symptomatic intracerebral haemorrhage after successful mechanical thrombectomy. *Clin Radiol*. 2021;76(4):316.e9-316.e18.
8. Ma C, Xu D, Hui Q, Gao X, Peng M. Quantitative Intracerebral Iodine Extravasation in Risk Stratification for Intracranial Hemorrhage in Patients with Acute Ischemic Stroke. *AJNR Am J Neuroradiol*. 2022;43(11):1589-1596.
9. Renú A, Amaro S, Laredo C, Román LS, Llull L, Lopez A, et al. Relevance of blood-brain barrier disruption after endovascular treatment of ischemic stroke: dual-energy computed tomographic study. *Stroke*. 2015;46:673-679.
10. Tijssen MP, Hofman PA, Stadler AA, van Zwam W, de Graaf R, van Oostenbrugge RJ, et al. The role of dual energy CT in differentiating between brain haemorrhage and contrast medium after mechanical revascularisation in acute ischaemic stroke. *Eur Radiol*. 2014;24(4):834-840.
11. Zaouak Y, Sadeghi N, Sarbu N, Ligot N, Lubicz B. Differentiation between Cerebral Hemorrhage and Contrast Extravasation Using Dual Energy Computed Tomography after Intra-Arterial Neuro Interventional Procedures. *J Belg Soc Radiol*. 2020;104(1):70.

Supplementary figure 1: Representative case.


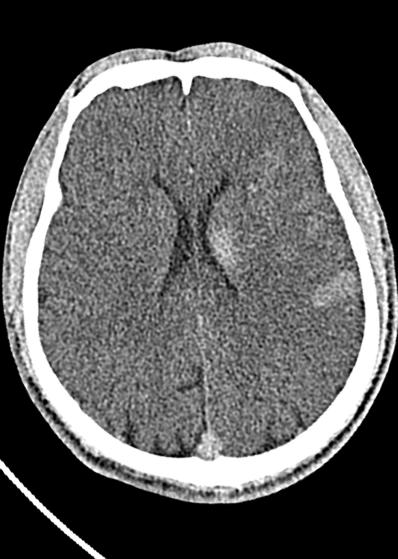

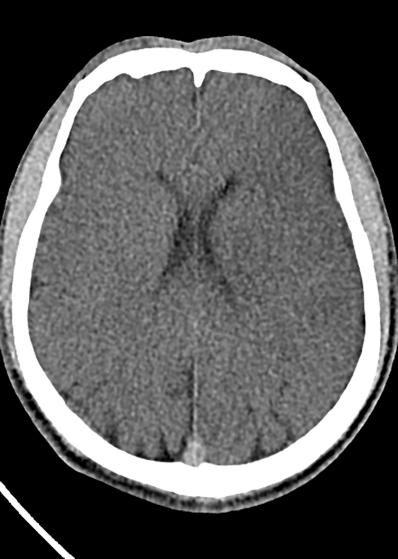

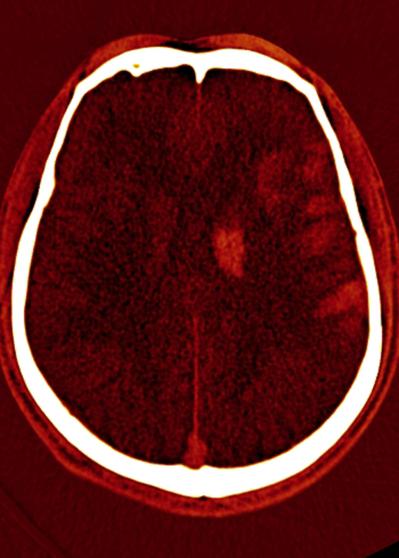


**c**

**b**

**a**


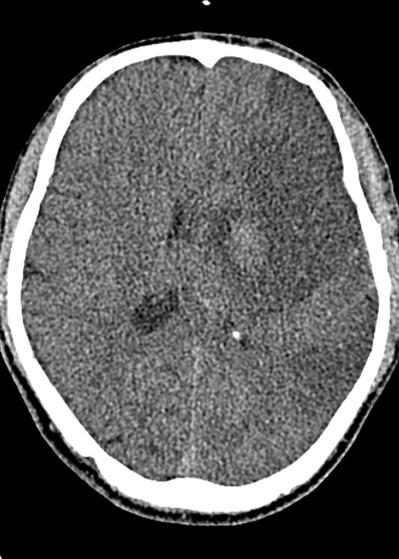

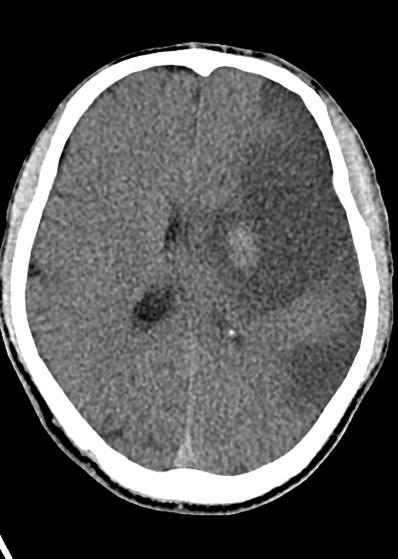

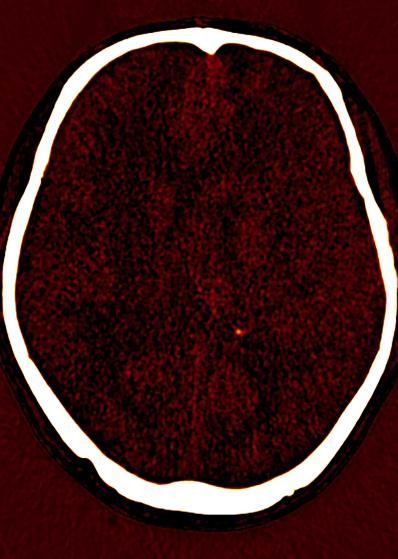


**f**

**e**

**d**

A 47 years old man, was admitted to hospital due to left middle cerebral artery M1 segment occlusion. The patient was immediately treated with endovascular thrombectomy and got complete recanalization. DECT within 2 hours after thrombectomy demonstrated parenchymal hyperdensity in the left basal ganglia, frontal and temporal lobe with infarction (a), without hemorrhage on the virtual noncontrast DECT (b) and with contrast staining on the iodine overlay map (c). Follow-up DECT within 12-24 hours demonstrated parenchymal hyperdensity in the left basal ganglia with evident massive infarction (d), with hemorrhage on the virtual noncontrast DECT (e) and almost without contrast staining on the iodine overlay map (f). The mRS score was 4 at discharge.
